# Remotely Delivered Video Interaction Guidance for Families of Children With an Intellectual Disability Referred to Specialist Mental Health Services: Protocol for a Feasibility Randomized Controlled Trial

**DOI:** 10.2196/54619

**Published:** 2024-12-05

**Authors:** Charmaine Kohn, Lauren Turner, Zhixing Yang, Michael Absoud, Angela Casbard, Manuel Gomes, Gemma Grant, Angela Hassiotis, Eilis Kennedy, Sophie Levitt, Rachel McNamara, Elizabeth Randell, Vasiliki Totsika

**Affiliations:** 1 The Tavistock and Portman NHS Foundation Trust London United Kingdom; 2 University College London London United Kingdom; 3 Evelina London Children’s Hospital London United Kingdom; 4 Centre for Trials Research Cardiff University Cardiff United Kingdom; 5 Challenging Behaviour Foundation Chatham United Kingdom; 6 Brighter Futures for Children Reading United Kingdom

**Keywords:** learning disability, intellectual disability, ID, child mental health, challenging behavior, family therapy, parent-child relations, parenting

## Abstract

**Background:**

Children with an intellectual disability (ID) are 3-4 times more likely to present with behaviors that challenge and mental health problems than typically developing children. Parenting and the quality of parent-child relationships are risk factors for these families. The COVID-19 pandemic further exacerbated difficulties, leading to an increase in child mental health problems and behaviors that challenge, a deterioration in parental mental health, and further strain on family relationships. Remote family interventions could be an effective solution for both families and specialist mental health services. Video interaction guidance (VIG) has shown promise for improving child mental health. However, it is unclear whether it is widely acceptable to families and feasible to implement across specialist child mental health services.

**Objective:**

This randomized controlled trial aims to evaluate the feasibility of delivering VIG as a remote intervention for parents of children aged 6-12 years with ID who have been referred to specialist mental health services.

**Methods:**

The study will be undertaken across 5-7 National Health Service specialist mental health services in England, involving 50 participants randomly allocated on a 1:1 basis to either the intervention group (receiving remote VIG) or the treatment-as-usual (TAU) group. The intervention group will engage in 3-5 cycles of VIG delivered remotely over 12 weeks. The primary feasibility outcomes include the recruitment rate, retention at 6-month follow-up, and VIG cycle completion rate. The secondary outcomes will assess the acceptability of VIG and the feasibility of remote implementation, including fidelity to the intervention protocol. Data will be gathered through online surveys and telephone interviews at baseline, 3 months, and 6 months. Feasibility outcomes will be summarized using descriptive statistics, while thematic analysis will be applied to qualitative data from semistructured interviews with participants, VIG practitioners, and service managers. An embedded process evaluation will explore barriers and facilitators to engagement with VIG, and a parallel health economics evaluation will assess the feasibility of capturing service use data and intervention costs.

**Results:**

The trial was open to recruitment between December 2022 and March 2024. The first results should be available in 2025.

**Conclusions:**

The study is the first randomized evaluation of VIG as offered to parents of children with ID who have been referred to specialist mental health settings. The outcomes from this feasibility trial will inform the decision to proceed with a definitive trial, using a traffic light system to evaluate recruitment, retention, and VIG completion rates alongside qualitative insights and economic evaluations.

**Trial Registration:**

ISRCTN Registry ISRCTN13171328; http://www.isrctn.com/ISRCTN13171328

**International Registered Report Identifier (IRRID):**

DERR1-10.2196/54619

## Introduction

### Background

Around 300,000 children in England have an intellectual disability (ID) [1]. ID often co-occurs with other neurodevelopmental conditions, such as autism. Children with IDs are 3-4 times more likely to exhibit challenging behaviors and mental health problems compared with typically developing children. By the age of 5 years, up to 88% of these children show clinical levels of hyperactivity, conduct problems, and emotional issues [2-5]. By mid-childhood (ages 11-12 years), challenging behaviors and mental health problems remain significantly higher in children with IDs compared with typically developing children [4,6].

The term “behaviors that challenge” is specifically used in relation to individuals with IDs or other groups who may have difficulty communicating. It refers to behaviors that, due to their frequency or intensity, place the individual or those around them at risk of harm or exclusion [7,8]. The term emphasizes that these behaviors pose a challenge to services, rather than being inherent problems within individuals with IDs. The term encompasses a diverse range of phenotypically different behaviors (eg, self-injury, aggression), defined by their impact on the environment. In ID, there is evidence of a close association between these behaviors and mental health problems [9].

Parenting and the quality of the parent-child relationship are risk factors for challenging behaviors and mental health problems in these families [10-13]. Children with IDs are at higher risk of experiencing negative parenting and poorer parent-child relationships [12]. The COVID-19 pandemic has further exacerbated these difficulties, leading to an increase in child mental health problems and challenging behaviors, a decline in parental mental health, and additional strain on family relationships due to service disruptions and restrictions [14].

Children with IDs who are suspected of having mental health problems or who exhibit challenging behaviors are typically referred to specialist mental health services. These services are increasingly under strain, often resulting in long wait times for families seeking therapy and support. Remotely delivered family interventions could offer an effective solution for both families and specialist mental health services [15]. In the United Kingdom, there is a growing impetus to adopt digital interventions to alleviate pressure on child mental health services, especially in the postpandemic context [16].

Specialist Child and Adolescent Mental Health Services (CAMHS) often provide either group- or individual-based programs. However, evidence shows very low access rates [17] and high dropout rates, often due to a perceived poor fit with family needs [18]. Parents tend to prefer personalized support that is flexibly delivered in their own environment and tailored to their family’s specific needs [19,20].

A comprehensive review of the literature on the effectiveness and acceptability of online-delivered family or parenting interventions highlights a growing body of research showing promising outcomes and generally positive user perceptions [21-23]. Numerous studies have demonstrated the effectiveness of these interventions in improving various aspects of family functioning, parent-child relationships, and child behavior [21,22]. Meta-analyses and systematic reviews consistently demonstrate significant improvements in parenting skills, parent-child interactions, and child behavior problems through online interventions [23]. Furthermore, online interventions targeting specific issues such as parental stress, child developmental delays, or behavioral disorders have shown promising results [24,25].

Overall, users tend to find online family or parenting interventions highly acceptable due to their accessibility, convenience, and flexibility. Parents value the ability to access support and resources from the comfort of their homes, eliminating the need for travel or scheduled appointments [22,26]. Online platforms often provide a variety of multimedia resources, interactive tools, and peer support networks, which enhance user engagement and satisfaction [23,25]. However, challenges such as limited internet access, low technological literacy, and concerns about privacy and confidentiality can affect the acceptability of online interventions for some families, particularly those from disadvantaged backgrounds [21,23,26]. Tailoring interventions to meet the needs and preferences of diverse families, offering clear instructions and technical support, and addressing privacy and security concerns are crucial for improving the acceptability and effectiveness of online family interventions [22,24].

In addition to positive service user feedback, staff who developed expertise with remote interventions during the pandemic are eager to integrate them into regular service offerings. This could improve access, reduce waiting times, and lower nonattendance rates [27].

### Video Interaction Guidance

Video feedback interventions have shown promise in improving child mental health [28]. These interventions typically involve using videotaped parent-child interactions as a therapeutic tool [29]. Video interaction guidance (VIG) is a widely used video feedback intervention in services across the United Kingdom. VIG is a brief, personalized, strengths-based intervention that focuses on successful moments of parent-child interaction as a key therapeutic tool [29,30]. The theoretical foundation of VIG is rooted in Colwyn Trevarthen’s [29-32] theory of intersubjectivity, which describes the development of shared understanding between a parent and an infant through the parent’s responses to the infant’s communicative cues. A key proposition of this theory is that positive communicative interactions are fostered when the parent is attentive and responds to the child’s communicative attempts in an attuned manner [31,32]. Consistently doing this allows the dyad to progress from attuned communication to “mediated learning” [31,32]. This concept, derived from Vygotsky’s [33] work, suggests that during parent-child interactions, the parent must provide the right amount of “scaffolding” to help the child progress independently. Too little support may cause the child to fail, while too much support can prevent learning. The Principles of Attuned Interaction and Guidance (Textbox 1; [34]) that underpin VIG propose that children who feel listened to by their parents are more likely to follow parental instructions. When parents provide a consistent “foundation of love, play, and work,” they are more likely to find it easier to manage problematic behavior [29]. Furthermore, the clinician’s role and the recording of sessions enable parents to gain an objective perspective on their interactions with their child, offering an opportunity to more clearly observe their communication patterns, emotional responses, and parenting strategies [30]. As the clinician and parent engage in a shared review of the parent-child interaction, focusing on strengths and areas of competence, the parent gains deeper insights into their parenting practices and develops self-reflection skills [30]. By offering personalized guidance and support, the clinician mirrors the experience of an attuned and collaborative relationship for the parent [30]. Therefore, VIG is an intervention that primarily aims to enhance attuned interactions between the 2 communication partners, which may lead to improvements in child behavior problems through more positive interactions and relationships.

**AVIGuk (Association of Video Interaction Guidance UK) principles of attuned interaction and guidance.**

**1. Being attentive**

- Looking interested with a friendly posture
- Giving time and *space* to other
- Turning toward
- Wondering about what they are doing, thinking, or feeling
- Enjoying watching the other

**2. Encouraging initiatives**

- *Waiting*
- Listening actively
- Showing emotional warmth through interaction
- Using friendly or playful interaction as appropriate
- *Naming* what the child is doing, might be thinking or feeling
- Naming what you are doing, thinking, or feeling
- *Looking for initiatives*

**3. Receiving initiatives**

- Showing you have heard, noticed the other’s initiative
- Receiving with body language
- Being friendly or playful as appropriate
- Returning eye contact, smiling, and nodding in response
- Receiving what the other is saying or doing with words
- Repeating/using the other’s words or phrases

**4. Developing attuned interactions**

- *Receiving and then responding*
- Checking the other is understanding you
- Waiting attentively for your turn
- Having fun
- Giving a second (and further) turn on the same topic
- Giving and taking short turns
- Contributing to interaction/activity equally
- Co-operating—helping each other

**5. Guiding**

- Scaffolding
- Saying “no” in the “yes” cycle (attuned limit setting)
- Extending, building on the other’s response
- Judging the amount of support required and adjusting
- Giving information when needed
- Providing help when needed
- Offering choices that the other can understand
- Making suggestions that the other can follow

**6. Deepening discussion**

- Supporting goal setting
- Sharing viewpoints
- Collaborative discussion and problem-solving
- Naming differences of opinion
- Investigating the intentions behind the words
- Naming contradictions/conflicts (real or potential)
- Reaching new shared understandings
- Managing conflict (back to being attentive and receiving initiatives with the aim of restoring attuned interactions)

Note: Principles taken from [34].

Systematic reviews have identified significant improvements in the quality of parent-child interactions and enhancements in child attachment following video feedback interventions [28,35,36]. Despite being recommended by the National Institute for Health and Care Excellence (NICE), empirical evidence regarding the effectiveness of video feedback interventions, particularly VIG, remains notably sparse. NICE guidelines advocate for the use of video feedback interventions for preschoolers exhibiting social-emotional problems [37] and for children experiencing attachment difficulties [38]. However, the existing evidence does not specifically pertain to VIG, and to date, no efficacy trials focusing solely on VIG have been conducted. Furthermore, the available evidence lacks specificity regarding child behavioral and mental health concerns. Nevertheless, insights from research into risk mechanisms suggest that video feedback interventions may provide direct benefits by enhancing parent-child relationships and indirect benefits by addressing child mental health issues and challenging behaviors [10-13]. Additionally, preliminary findings suggest that video feedback interventions have the potential to alter adults’ perceptions of their relationship with a child who has an ID and presents challenging behaviors [39]. It is noteworthy that while NICE guidelines advocate for video feedback interventions in certain contexts, such as for children and young adults with autism [40], VIG is not specifically mentioned as a potential intervention for parents or caregivers of older children with IDs. This population may require tailored interventions that specifically address their unique needs. In the context of ID, there is a focus on interventions that directly reduce challenging behaviors (eg, parenting courses) [41]. It is important to highlight NICE’s recommendation for further research involving children with IDs, specifically calling for studies that examine community-based interventions aimed at reducing the frequency and severity of challenging behaviors [41]. Interventions designed to support communication and interaction between young people with IDs and their families are crucial. Interpersonal interventions, such as VIG, could be highly beneficial due to their focus on enhancing communication between parents and children, as well as their potential to indirectly impact other child outcomes, such as challenging behaviors [42].

Specialist mental health services are beginning to offer VIG to families of children with IDs and comorbid conditions aged 6 years and older. Although VIG shows promise and is utilized in some services, it remains unclear whether it is widely acceptable to families and feasible to implement across specialist services. VIG has never been evaluated in specialist mental health services, and only 2 small studies have been conducted to date: a pilot trial (N=31) involving preterm neonates [43] and an uncontrolled feasibility study (N=19) with infants [44]. Although neither of these studies included families of children with IDs, Barlow et al’s [43] pilot randomized controlled trial (RCT) investigated the application of VIG with preterm infants, finding improvements in parent-baby interaction and parental sensitivity. Improvements can be attributed to the key therapeutic components of VIG, such as attuned communication, reflective dialog, positive reinforcement, and the promotion of sensitive and responsive caregiving behaviors. Similarly, the feasibility study by Chakkalackal et al [44] in infant mental health supports these findings, indicating improvements in parental insight and sensitivity; however, the primary outcome of that study was participant engagement and recruitment. Although neither study directly assessed the efficacy or effectiveness of VIG, they do provide preliminary evidence for the types of outcomes that could be observed when VIG is used within a health setting. Furthermore, both studies included only immediate follow-up assessments [43,44], whereas VIG outcomes should be measurable beyond this initial phase [28,36]. In particular, any changes should be demonstrated in the medium term (eg, at a 6-month follow-up), as longer-term impacts on child outcomes in this population are unlikely to be sustained [45].

A recent study indicated that the key therapeutic mechanisms of VIG are present at similar levels when offered remotely compared with face-to-face [46]. Specifically, the study compared face-to-face and online interactions between VIG practitioners and parents, finding no significant differences in the levels of warmth, responsiveness from both clients and clinicians, or the balance in their interactions [46]. Therapists who used VIG remotely during the COVID-19 lockdown offered insights into how to enhance remote delivery, with 82% expressing a desire to continue providing VIG remotely after the pandemic [46]. While the “Zoom or Room” study provided encouraging evidence of the efficacy of online VIG, it is important to acknowledge the limited literature on the impact of delivering VIG remotely compared with face-to-face interactions. While online interventions offer advantages in terms of accessibility and flexibility, general concerns persist regarding the depth of connection, quality of communication, and therapeutic alliance developed within online therapeutic interventions compared with traditional face-to-face interactions [47,48]. Furthermore, the “Zoom or Room” study was conducted during the COVID-19 pandemic, a time when online therapies were in high demand and served as a primary means of social interaction. This context may have influenced participant attitudes and engagement, potentially biasing the findings. Given the limited evidence regarding the effectiveness of VIG delivered remotely outside of a pandemic context, further investigation is warranted to assess the feasibility of online VIG in a postpandemic environment, where the demand for and reliance on online interventions may vary.

A study is therefore needed to determine the feasibility of an RCT evaluating remotely delivered VIG for parents of children with IDs referred to specialist child mental health services. The study will focus on assessing the acceptability of remote VIG in this population, as indicated by both recruitment rates and engagement with the intervention. It will also investigate whether parents referred to specialist services for child mental health problems or challenging behaviors prefer support focused solely on the child. Additionally, the study will assess whether an appropriate primary outcome for a definitive evaluation can be identified, based on stakeholder perspectives regarding perceived impacts and the adequacy of child outcome measures.

### Objective

This study aims to determine the feasibility of an RCT evaluating remotely delivered VIG to parents of children aged 6-12 years with IDs who have been referred to specialist child mental health services. The primary feasibility objectives of the study are to assess (1) the participant recruitment rate, (2) study retention at the 6-month follow-up, and (3) the rate of VIG completion (defined as completing 3 out of a maximum of 5 VIG cycles). The study’s secondary objectives are as follows: (1) to assess the completeness of outcome measures; (2) to investigate the acceptability of VIG among parents and practitioners, including identifying barriers and facilitators to the remote offering and uptake of VIG; (3) to evaluate the feasibility of remote implementation, which includes measuring VIG fidelity, perceived effectiveness, necessary adaptations, and any unintended implementation failures; and (4) to provide preliminary evidence regarding service use assessments and the costs associated with the remote delivery of VIG in specialist mental health services. Additionally, the study will use a traffic light system [49] to evaluate the feasibility of progressing to a definitive trial.

## Methods

### Study Design

**Figure 1:**
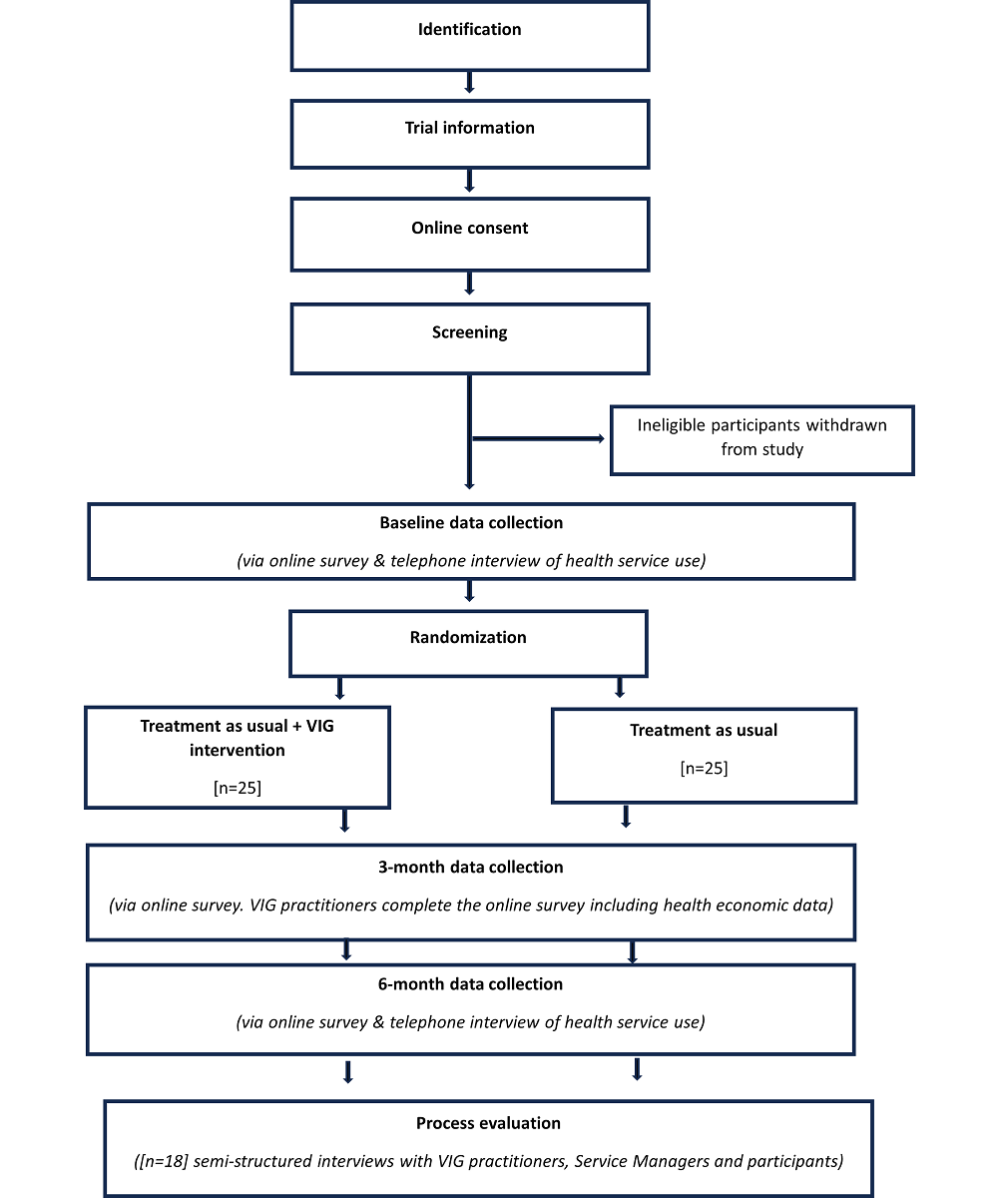
Flowchart of participants through the VIG-LD feasibility trial with 2 randomized groups. LD: learning disability; VIG: video interaction guidance.

This feasibility RCT will involve 50 parents, who will be allocated on a 1:1 basis to either the intervention or treatment as usual (TAU), along with an embedded process evaluation. Additionally, service use data will be collected for cost comparison as part of a parallel feasibility economic evaluation (Figure 1).

### Study Setting

The study will be conducted in National Health Service (NHS) specialist mental health services. Children with an ID are referred to these services due to high levels of challenging behaviors or if they are suspected of having mental health problems. There are generally 2 types of specialist mental health services. The first type includes CAMHS that specifically caters to children with IDs, known in the United Kingdom as CAMHS Learning Disability (LD), or those that have a neurodevelopmental pathway. The second type consists of specialist pediatric neurodevelopmental services that include a pathway for addressing a child’s behavioral and mental health problems. If the service includes a diagnostic pathway, children may be referred for an assessment of suspected neurodevelopmental disabilities. In these cases, diagnostic assessments may lead to a referral to the service’s mental health pathway if mental health problems or challenging behaviors are identified during the referral process or the initial assessment. Specialist CAMHS services typically accept children from the age of 6 years, whereas specialist pediatric neurodevelopmental services accommodate a broader age range.

The study will be a multicenter investigation, with 5-7 sites anticipated to participate in England. Each site will carry out the same activities, including recruitment for the study, providing VIG to the intervention group, and offering treatment as usual (TAU) to both the intervention and comparison groups. Some sites may already have 1 or more trained VIG therapists who incorporate VIG into their therapeutic pathways, while others may not have any trained VIG therapists and may not include VIG in their standard offerings.

### Ethics Approval

The study received approval from independent reviewers of the London South East Ethics Committee (22/LO/0819).

### Participants and Eligibility Criteria

The study population comprises parents of children aged 6-12 years with IDs who have been referred to specialist mental health services. The lower age limit was chosen to align with the typical age at which children are referred to these services (as outlined in the study settings above), while the upper age limit reflects the age at which the UK NICE recommends that parenting support be provided [50]. Eligible participants will be identified from the waiting lists of specialist mental health services, including both new and existing referrals. They will be screened according to the inclusion and exclusion criteria outlined in Textbox 2.

**Participant inclusion and exclusion criteria.**

1. **Inclusion criteria**
  - The parent is at least 18 years of age.
  - The parent has a child who is aged between 6 and 12 years (up to 1 day before the 13th birthday on screening day).
  - The parent is the child’s biological, foster, adoptive or stepparent, or any other caregiver who lives with the child.
  - The child has an administratively defined intellectual disability: that is, an administrative label within the education, health, or social care system identifying intellectual disability; or as eligible for neurodevelopmental services; or a diagnosis (learning/intellectual disability or [global] developmental delay for younger children). The child may be diagnosed with additional conditions (eg, Down syndrome) or co-occurring neurodevelopmental conditions (autism). Children with co-occurring conditions are eligible.
  - The child has a composite score of <80 on the Vineland Adaptive Behavior Scales (Vineland-3 30), indicating significant developmental delay.
  - The child has been referred to a specialist child mental health service (new or existing referral).
2. **Exclusion criteria**
  - Another sibling participates in the trial.
  - The parent is receiving another video feedback intervention (Video-Feedback Intervention to Promote Positive Parenting (VIPP), Video-Feedback Intervention to Promote Positive Parenting and Sensitive Discipline (VIPP-SD), Marte Meo, Video Parent-Child Interaction, Paediatric Autism Communication Therapy) either remotely or in person.
  - The child lives with the parent <50% of the time or is in a 24-hour residential placement (inpatient unit or residential school).
  - The family is under active family court proceedings.

### Recruitment

#### Identification of Potential Participants for the RCT

During the recruitment stage, potential study participants will be identified through 2 methods: either directly by clinicians during routine clinical contact or via site mail-outs that utilize the service’s referral or waiting or active caseload lists. In both cases, eligible participants will receive a summary of the study. For cases referred by a participant identification center, clinicians will receive guidance information. Additionally, participant identification centers will be provided with supporting information about the study in case they wish to refer a parent of a suitable child.

Interested parents who receive the study information will contact the researcher directly. The researcher will respond to all expressions of interest via email or SMS text message, offering to discuss the study in more detail. If the potential participant does not respond after 3 attempts, no further contact will be made. For those who do respond, a time will be scheduled to discuss the study and review the participant information sheet (PIS).

#### Identification of Process Evaluation Interview Participants

The study will use random sampling to identify potential interview participants from among the parents who consented and were deemed eligible for the trial. We will ensure a balanced sample across variables such as group allocation and site. Clinical service staff who have provided VIG to at least one family will be approached for an interview, with a minimum of 1 practitioner recruited per site. Additionally, all service managers will be invited to participate in an interview.

### Consent

Before any data collection, including screening, written informed consent will be obtained from all study participants through an online form via Qualtrics (Qualtrics International Inc.). Participants will first receive the PIS when they express interest in the study. They will then discuss the PIS with a researcher to ensure full understanding. The PIS includes information about the intervention, the research tasks required, the randomization process, details of ethical approval, and how any data will be managed and stored. All personal information will be stored securely within the study host organization, and anonymized data will be transferred from clinical staff involved in VIG delivery or collaborating organizations. At the end of the meeting, participants will receive a personalized link to the informed consent form via email. The consent form was designed in accordance with the joint statement on e-consent from the Health Research Authority and the Medicines and Healthcare Regulatory Agency [51]. If participants are unable to complete the consent process in Qualtrics, an editable offline version of the consent form will be provided, which they can sign by hand and return to the research team.

Consenting and eligible participants will receive a child notification letter that explains the purpose and process of the study to the children. This letter will include information on how children can contact the trial manager if they wish to do so.

For participants in the process evaluation interviews, which include both clinical service staff and parents, a separate consent form will be obtained alongside a PIS.

### Screening of Potential Participants for RCT Eligibility

The screening assessment will be conducted over the phone or via videoconference by a researcher using a standardized form. The Vineland Adaptive Behavior Scales (Vineland-3; 52) will be completed during the same meeting or in a subsequent appointment if necessary. Once screening is complete and eligibility is confirmed, participants will receive a personalized link to the first data collection questionnaire (baseline).

### Randomization

Randomization will occur after participants have provided consent, had their eligibility confirmed through the screening process, and completed baseline data collection. Participants will be randomly assigned on a 1:1 basis to 1 of the 2 study arms (VIG + TAU vs TAU). Random block allocation stratification techniques will be used to ensure a balanced sample across variables such as group allocation and site.

### Sample Size and Power

As this study is a feasibility RCT, a power calculation was not used to estimate the target sample size. Instead, we examined similar studies [44,52] to determine an appropriate recruitment rate for the study. Based on this analysis, we expect to recruit at least 50 participants from approximately 100 families invited to participate. The feasibility of recruitment will be assessed using a hypothesis-testing approach [53]. This assessment is 1 of the 3 primary research objectives, with a green signal indicating a recruitment rate of 50% of eligible families, amber indicating a rate of 35%-50%, and red indicating less than 35%. To achieve 90% power and a 5% 1-sided α, 97 families will need to be approached.

### Intervention

#### Video Interaction Guidance

The VIG intervention involves the practitioner capturing a short video of the parent interacting with their child during the first meeting, which lasts approximately 20 minutes. In the subsequent meeting, known as the shared review, the practitioner and parent watch selected moments from the video where the interaction between parent and child is particularly positive, based on the parent’s successful communication [30]. The practitioner guides the parent in identifying these moments and highlights their contributions to successful, attuned interactions.

The VIG practitioner facilitates the video recording of a parent-child interaction during an initial meeting with the parent before the recording date. In this meeting, the parent identifies their goals for the VIG intervention. The practitioner assists the parent in recognizing when these goals are being met, even if only briefly, and they discuss activities where such moments might occur. The parent is encouraged to engage in 1 of these activities during the recorded interaction with their child. This approach ensures that the video captures a particularly positive interaction, rather than just typical free play. The chosen activity should be enjoyable and comfortable for both the parent and child. Typically, VIG practitioners film for 5-10 minutes.

For this study, remote meetings are conducted via familiar video calling platforms such as Microsoft Teams (Microsoft Corporation) or Zoom (Zoom Video Communications, Inc.); however, the initial meeting may occur face-to-face if both the practitioner and parent agree. The practitioner helps the parent and child set up the activity, ensuring good visibility and audio. Afterward, they start the recording and turn off their own camera and microphone. They monitor the recording, intervening if the child or parent becomes upset, with the goal of maintaining a positive interaction. Afterward, the practitioner analyzes the recording using principles of attuned interaction, identifying short clips of the most attuned moments for review with the parent. Shared review meetings typically last between 30 minutes and 1 hour. Each recording and review session constitutes 1 cycle of VIG intervention, with 3-5 cycles making up a full intervention. Typically, 3 cycles are offered, with the option to request 2 additional cycles. For the purposes of this study, the maximum intervention duration is 12 weeks, which includes an introductory meeting and a review session. Parents in the VIG group may also receive additional support, either face-to-face or remotely, as provided by their service.

#### Treatment as Usual

Participants allocated to TAU will serve as the comparison group and receive the standard clinical care provided by the specialist mental health service. In parallel with the feasibility RCT, a survey of specialist mental health services across the United Kingdom will be conducted to provide an in-depth description of how widely these services offer video feedback and other interventions, at either the referral/waiting list stage or the active caseload phase.

### Data Collection and Analysis

#### Overview

The primary aim of the study is to assess the feasibility of conducting a full trial of VIG-LD using the current design, with potential modifications (and, if so, which ones), or to determine whether a full trial should not be pursued. Below, we describe the analysis approach for each outcome and outline the traffic light criteria to be used where applicable. Table 1 presents the primary outcomes and the associated progression criteria.

**Table 1:** Primary outcomes and traffic light progression criteria.

Outcome	Definition
Recruitment rate	The number of participants eligible to participate among those undergoing formal screening and the number of those screened who are randomized. The desired (green light) criterion to be met is that at least 50% of eligible parents participate, that is, 50 of the 97 eligible. The traffic light system is set to red if fewer than 35% agree to randomization.
Study retention rate (participants at 6 months)	The number/proportion of randomized participants who have at least one parent questionnaire completed at the 6-month follow-up among all participants who consented to participate. The number of participants who at the 6-month follow-up provide useable data on Developmental Behaviour Checklist-2 among those randomized. The desired (green light) criterion to be met is that at least 70% of recruited participants are retained at the 6-month follow-up. The traffic light system is set to red if fewer than 60% are retained.
VIG^a^ completion/adherence	The number/proportion of participants who complete the recommended amount of the VIG-LD^b^ intervention (3 cycles) among all participants randomized in the VIG intervention group. The desired (green light) criterion to be met is that at least 80% of participants receive 3 VIG cycles. VIG cycle completion is defined as 1 meeting to take a video and 1 meeting to go through the shared review. A traffic light system is set to red if fewer than 65% receive 3 VIG cycles.

^a^VIG: video interaction guidance.

^b^LD: learning disability.

#### Primary Outcomes

##### Summary

The primary outcomes being assessed include the feasibility of delivering and evaluating remote VIG, the participant recruitment rate, study retention at the 6-month follow-up, and the VIG completion rate. The primary outcomes that will be analyzed are described in the subsequent sections.

##### Recruitment

The study will record the number of referrals directly from clinicians, the number of individuals on mailing lists who were emailed study information, the number of interested parents undergoing screening, the number deemed eligible or ineligible (with reasons for ineligibility), the number who provide consent, the number randomized, and the reasons for refusing randomization. The recruitment rate will be estimated based on the number of parents found eligible for the trial after formal screening and the number of those eligible who are randomized. The desired criterion is that at least 50% of eligible parents are randomized. However, the traffic light system sets 35% as the threshold for the amber zone, meaning 34 out of 97 eligible parents would need to be randomized. If recruitment falls within the amber zone but exceeds the critical value of 42 (54%), minor adjustments will be needed to improve recruitment. If recruitment falls below 42, major changes will be necessary.

##### Study Retention

This will be measured as the percentage of participants who have completed at least one questionnaire at the 6-month follow-up among all those who consented to participate. The criterion to be met is that at least 70% of randomized participants are retained at the 6-month follow-up (60%-69% falls within the amber zone and <60% within the red zone). See Table 2 for a full list of questionnaire measures.

**Table 2:** Full list of data collection measures completed within the trial.

Measure	Screening	Baseline	3-Month follow-up	6-Month follow-up
VABS-3^a^ (socialization and communication subsections only at the 6-month follow-up)	✓			✓
PHQ-4^b^		✓	✓	✓
PSOC^c^		✓	✓	✓
CPRS^d^		✓	✓	✓
APQ^e^		✓	✓	✓
DBC-2^f^		✓		✓
CA-SUS^g^		✓		✓
ESQ^h^			✓	
GBO^i^ (VIG^j^ therapist only)			✓	
Process evaluation semistructured interview				✓

^a^VABS-3: Vineland Adaptive Behaviour Scale (Vineland–3) [54].

^b^PHQ-4: Patient Health Questionnaire-4 [55].

^c^PSOC: Parenting Sence of Competence Scale [56].

^d^CPRS: Child-Parent Relationship Scale [57].

^e^APQ: Alabama Parenting Questionnaire [58].

^f^DBC-2: Developmental Behaviour Checklist-2 [59]

^g^CA-SUS: Child and Adolescent Service Use Survey [60].

^h^ESQ: Experience of Service Questionnaire [61].

^i^GBO: Goal Based Outcomes [62].

^j^VIG: video interaction guidance.

##### VIG Completion

This will be measured by the number of participants in the intervention group who complete at least three VIG cycles. The criterion to be met is that at least 80% of participants receive 3 VIG cycles (65%-79% fall within the amber zone and <65% within the red zone). A VIG cycle is defined as 1 meeting to record a parent-child interaction and 1 meeting to view and discuss edited clips of the most attuned moments during the shared review.

#### Secondary Outcomes

##### Overview of Secondary Outcomes and Health Economic Evaluation

Secondary outcomes will include the completeness of outcome measures (ie, usable items), acceptability, barriers and facilitators to engaging with remote VIG for both parents and VIG practitioners, and the feasibility of remote implementation (both VIG and study processes). This will encompass aspects such as VIG fidelity, perceived effectiveness, potential adaptations, and any unintended implementation failures. The health economic evaluation will assess the feasibility of measuring health and social care services utilized by children with IDs whose parents participate in either the treatment as usual or VIG groups. This evaluation will include information on medications used by both the study participants and their children with IDs. Additionally, the health economics evaluation will aim to estimate the total cost of delivering remote VIG and assess how these costs compare with those of providing treatment as usual.

Qualitative data will be collected from semistructured interviews with participants, VIG practitioners, and service managers. These interviews will focus on discussions about participants’ experiences engaging with the research tasks, including what they found favorable or unfavorable about the research process and the intervention itself. Additionally, participants will be invited to share suggestions for potential improvements in both the research methodology and the delivery of the intervention.

Health economics data will be collected through telephone-based interviews with participants, conducted at baseline and at the 6-month follow-up, as well as through an online survey completed by clinical staff at the 3-month follow-up. Health and social care utilization will be captured via the telephone interview by adapting the Child and Adolescent Service Use Schedule (CA-SUS) for this study. This questionnaire has been previously adapted for this population [14] and includes items related to primary and secondary care services, as well as medications used over the past 6 months. Service use will be costed using unit costs from the Personal Social Services Research Unit, while medication costs will be determined using the British National Formulary. The CA-SUS will aim to collect all relevant health and social care utilization data, including any service use components that may overlap between VIG and TAU interventions. Costs associated with the delivery of VIG will be gathered through an online survey completed by staff. This survey will collect data on staff salary banding and the number of hours spent on delivery-related tasks.

The economic evaluation will be conducted from a health and social care perspective; therefore, recruitment costs will be excluded.

The secondary outcomes will be analyzed as described in the following sections.

##### Completeness of Outcome Measures

This will refer to the number of participants who provide usable data for each study measure, estimated separately at each time point. The definition of “usable” data will be based on having enough items to allow for the calculation of a total or subscale score. The percentage of participants who provided data on the DBC-2 [59] at the 6-month follow-up will be estimated among those recruited (likely the primary outcome in the final trial). A green signal for completeness will be defined as having 80%-100% usable data for DBC-2 scores. Any measure with less than 70% usable data will be reevaluated.

##### Acceptability of VIG

Qualitative data from interviews with parents, VIG practitioners, and service managers will be analyzed using framework analysis to assess the acceptability of VIG [63]. This analysis will also identify key barriers and facilitators to engaging with remote VIG.

##### Feasibility of VIG Remote Implementation—Intervention Fidelity

Fidelity will be measured using the VIG-Skills Development Scale (SDS) [64]. This tool provides a structured assessment of VIG skills and is typically used in training and reflective practice to ensure practitioners’ fidelity to the therapeutic model, which emphasizes a strengths-based and balanced approach. The VIG-SDS will be used to measure the fidelity of VIG implementation by reviewing practitioners’ selection of video clip moments during the shared review, which is the meeting between the VIG practitioner and the parent (see the “Intervention” section). A total of 25 VIG cycles will be selected from the 75-125 cycles likely generated during the study. Fidelity will be assessed based on the percentage of reviewed cycles achieving a VIG-SDS score that corresponds to the expected level for the practitioner’s training.

##### Feasibility of Remote Implementation—Intervention and Study Processes

Guided by the Medical Research Council framework for process evaluation [65], the analysis will integrate both bottom-up and top-down approaches to identify key themes emerging from the data across the process evaluation domains of context, implementation, and mechanisms of impact. Satisfaction with specialist mental health service input will be measured by summing the 9 items from the Experience of Service Questionnaire (ESQ) and comparing the results between the 2 groups [61].

##### Health Economics Data Analysis

The health economic analysis will adopt a health and social care perspective. Costs associated with VIG practitioners and other health and social care service usage will be calculated using unit costs from health and social care sources [66]. Health and social service utilization, as well as medication use, will be captured using the CA-SUS [60]. The total cost of remote VIG delivery will be estimated based on the amount of time VIG practitioners spend on delivery and the number of completed VIG sessions or cycles.

### Participant Compensation

A small monetary compensation, in the form of a gift voucher, will be provided to participants after each instance of data collection (screening, baseline, 3-month follow-up, 6-month follow-up, and qualitative interview).

### Patient and Public Involvement

During the development of the study protocol, interviews were conducted with parents and VIG practitioners who had experience with remote VIG. Two parents who had received VIG remotely through their Educational Psychology service were interviewed, and 1 family had experience with an LD CAMHS referral while also receiving remote VIG from Educational Psychology. Parents shared their experiences and reflected on the process and perceived impacts, which contributed to the draft logic model and helped researchers identify additional outcomes for measurement, such as parenting efficacy. Parents questioned whether the effects of VIG were sustained in the medium term and expressed a desire for support that focused more on the child. This raised concerns about the acceptability of a parent-focused therapy such as VIG within a specialist CAMHS setting. Consequently, acceptability was considered the primary outcome of the feasibility study.

Consultations were also conducted with several VIG practitioners experienced in delivering VIG remotely, some of whom had worked with families of children with IDs. They emphasized that VIG is highly feasible for these families, even in a remote setting, and expressed support for its adoption by more services to improve accessibility.

During the conduct of the study, a Parent Carer Advisory Group (PCAG) consisting of 10 parents of children with IDs will be established to provide guidance on study materials and develop a participant recruitment video explaining the study. The PCAG will advise on recruitment materials, processes, data analysis, and dissemination of content and strategy. Additionally, 2 parents from the group will contribute directly to the academic paper during the dissemination phase. The study’s approach to patient and public involvement is informed by Staniszewska et al [67].

### Data Management and Storage

Investigators and research staff will adhere to the Data Protection Act 2018 and General Data Protection Regulation (GDPR) guidelines for handling personal information throughout the study. Qualtrics, a secure and GDPR-compliant platform, will be utilized for online data collection. Personal information will be kept separate from other data, with participant IDs linked to pseudonyms stored in a separate location. Transcripts and verbatim quotes will be pseudonymized, and audio recordings will be deleted after transcription. All data will be stored securely with password protection on study host servers. Access to participant-identifying information will be restricted to designated research staff. Pseudonymized data will be transferred securely between NHS and collaborating organizations for analysis. Personal identifying information will be retained until the study’s completion, while pseudonymized data will be stored for at least 20 years. Participants will be informed of these procedures in the PIS.

### Safety Reporting

In the event that any adverse events or serious adverse events related to the intervention or research procedures occur, the chief investigator and study coordinator will be informed through the completion of an adverse event/serious adverse event form. All events will be reported immediately, and within 24 hours of becoming aware of the event, to the sponsor by the study team. All events will be assessed at each follow-up time point, and intervention delivery staff will be trained to report these directly to the study team at any time during the study.

## Results

The study recruitment period was planned from December 2022 to March 2024. Recruitment has now been completed and analyses are planned to be completed by November 2024. The first results are expected to be available in 2025.

## Discussion

### Expected Findings

The findings from this feasibility trial are expected to provide valuable information about the acceptability and feasibility of VIG for parents of children with IDs within specialist mental health services. As the first randomized evaluation of VIG in these settings and for this population, no hypotheses can be made regarding the level of acceptability of the intervention or the feasibility of its remote delivery and evaluation. Previous research has emphasized the value of video feedback interventions, such as VIG, in improving parent-child interactions, which are crucial for supporting the mental health and behavioral development of children with IDs [29,30]. However, a previous single-group feasibility study of VIG with a different population (parents of infants) faced difficulties with recruitment and retention, despite high levels of acceptability for VIG (which was delivered face-to-face) [44]. Therefore, conducting a feasibility RCT is a necessary step in determining feasibility, acceptability, and other parameters required before a definitive trial.

### Conclusions

This study represents the first randomized evaluation of VIG offered remotely to parents of children with IDs referred to specialist mental health settings. The findings regarding acceptability and feasibility will inform the design of a definitive randomized trial, should the progression criteria be met.

### Dissemination Plan

The results of this feasibility trial will be disseminated as noted in Textbox 3.

**Dissemination of the trial results.**

**1. Parent Carer Advisory Group Involvement**

The Parent Carer Advisory Group (PCAG) will play an integral role in the dissemination process. Members of the PCAG will coauthor academic papers to ensure that the findings are communicated in a manner that is accessible and relevant to families of children with intellectual disabilities.

**2. Academic Publications and Conference Presentations**

The study’s findings will be submitted for publication in peer-reviewed journals that focus on child mental health, intellectual disability, and digital health interventions. Additionally, efforts will be made to present the results at national and international conferences to engage with the broader academic and clinical communities.

**3. Funder’s Website and Public Engagement**

The trial outcomes will be published on the funder’s website to ensure accessibility for the public, including families, health care professionals, and other interested parties.

**4. Collaborations With Specialist Services**

The findings will be shared with the National Health Service specialist mental health services that participated in the trial. These services will receive tailored reports highlighting the practical implications of the study, which may guide the wider adoption of remote video interaction guidance within these settings.

## Abbreviations

CAMHS LD: Child and Adolescent Mental Health Services Learning Disability
CA-SUS: Child and Adolescent Service Use Schedule
DBC-2: Developmental Behaviour Checklist 2 Scale
ESQ: Experience of Service Questionnaire
GDPR: General Data Protection Regulation
ID: intellectual disability
NHS: National Health Service
NICE: National Institute for Health and Care Excellence
PCAG: Parent Carer Advisory Group
PIS: participant information sheet
RCT: randomized controlled trial
SDS: Skills Development Scale
VIG: video interaction guidance

## Data Availability

The final data sets from the trial will be available upon request, and any third-party requests for access will be reviewed by the chief investigator.
